# Supplementary material for: Identification and functional analysis of non-coding regulatory small RNA FenSr3 in Bacillus amyloliquefaciens LPB-18
Source: PeerJ. 2023 May 15;11:e15236. doi: 10.7717/peerj.15236 (PMC10194069; doi:10.7717/peerj.15236)
Supplement: Supplemental Information 4 [file peerj-11-15236-s004.zip › KO/CK-vs-T1_map/map05223.html]

KEGG PATHWAY: Non-small cell lung cancer - Reference pathway


|  |  |
| --- | --- |
| **Non-small cell lung cancer - Reference pathway** |  |

[
Pathway menu
| Organism menu
| Pathway entry
| Show description
| User data mapping
]

Lung cancer is a leading cause of cancer death among men and women in industrialized countries. Non-small-cell lung cancer (NSCLC) accounts for approximately 85% of lung cancer and represents a heterogeneous group of cancers, consisting mainly of squamous cell (SCC), adeno (AC) and large-cell carcinoma. Molecular mechanisms altered in NSCLC include activation of oncogenes, such as K-RAS, EGFR and EML4-ALK, and inactivation of tumorsuppressor genes, such as p53, p16INK4a, RAR-beta, and RASSF1. Point mutations within the K-RAS gene inactivate GTPase activity and the p21-RAS protein continuously transmits growth signals to the nucleus. Mutations or overexpression of EGFR leads to a proliferative advantage. EML4-ALK fusion leads to constitutive ALK activation, which causes cell proliferation, invasion, and inhibition of apoptosis. Inactivating mutation of p53 can lead to more rapid proliferation and reduced apoptosis. The protein encoded by the p16INK4a inhibits formation of CDK-cyclin-D complexes by competitive binding of CDK4 and CDK6. Loss of p16INK4a expression is a common feature of NSCLC. RAR-beta is a nuclear receptor that bears vitamin-A-dependent transcriptional activity. RASSF1A is able to form heterodimers with Nore-1, an RAS effector.Therefore loss of RASSF1A might shift the balance of RAS activity towards a growth-promoting effect.

|  |  |  |
| --- | --- | --- |
| Reference pathway | 184% 150% 122% 100% 82% 67% 55% | 图片下载 |
